# Endothelial cell dysfunction and cardiac hypertrophy in the STOX1 model of preeclampsia

**DOI:** 10.1038/srep19196

**Published:** 2016-01-13

**Authors:** Aurélien Ducat, Ludivine Doridot, Rosamaria Calicchio, Celine Méhats, Jean-Luc Vilotte, Johann Castille, Sandrine Barbaux, Betty Couderc, Sébastien Jacques, Franck Letourneur, Christophe Buffat, Fabien Le Grand, Paul Laissue, Francisco Miralles, Daniel Vaiman

**Affiliations:** 1 Inserm, U1016, Institut Cochin, Paris, France; 2 Cnrs, UMR8104, Paris, France; 3 Université Paris Descartes, Sorbonne Paris Cité, Paris, France; 4 DHU Risques et Grossesse, PRES Sorbonne Paris Cité, 53 avenue de l’observatoire, 75014 Paris; 5 Unité Mixtes de Recherche1313 Génétique Animale et Biologie Intégrative, Institut National de la Recherche Agronomique Jouy-en-Josas, France; 6 Genetics and Endocrine Oncology, Hôpital de la Conception Assistance PubliqueHopitaux de Marseille, Marseille, France; 7 Unidad de Genética. Grupo GENIUROS. Escuela de Medicina y Ciencias de la Salud. Universidad del Rosario. Bogotá, Colombia

## Abstract

Preeclampsia is a disease of pregnancy involving systemic endothelial dysfunction. However, cardiovascular consequences of preeclampsia are difficult to analyze in humans. The objective of the present study is to evaluate the cardiovascular dysfunction induced by preeclampsia by examining the endothelium of mice suffering of severe preeclampsia induced by STOX1 overexpression. Using Next Generation Sequencing on endothelial cells of mice carrying either transgenic or control embryos, we discovered significant alterations of gene networks involved in inflammation, cell cycle, and cardiac hypertrophy. In addition, the heart of the preeclamptic mice revealed cardiac hypertrophy associated with histological anomalies. Bioinformatics comparison of the networks of modified genes in the endothelial cells of the preeclamptic mice and HUVECs exposed to plasma from preeclamptic women identified striking similarities. The cardiovascular alterations in the pregnant mice are comparable to those endured by the cardiovascular system of preeclamptic women. The STOX1 mice could help to better understand the endothelial dysfunction in the context of preeclampsia, and guide the search for efficient therapies able to protect the maternal endothelium during the disease and its aftermath.

Preeclampsia is a complication of pregnancy characterized by gestational hypertension and proteinuria^1^. Preeclampsia affects ~5% of pregnant women leading to maternal an fetal morbidity and mortality^2^,^3^.

Endothelial dysfunction is a characteristic of preeclampsia^4^. Women affected by preeclampsia have also cardiac dysfunction, and cardiac hypertrophy^5^. Epidemiological data report an increased risk of cardiovascular diseases later in life in women who have had a preeclamptic pregnancy^6^.

We recently developed a mouse model of preeclampsia by transgenic overexpression of the transcription factor STOX1^7^. STOX1 was identified in Dutch families by a positional cloning approach, and its involvement in the pathophysiology of preeclampsia was inferred^8^. Crossing transgenic males with WT females induces a severe preeclamptic phenotype, with gestational hypertension, proteinuria, increased plasma levels of soluble Endoglin (sENG) and soluble FMS-like tyrosine kinase 1 (sFLT1), as well as kidney histological anomalies in line with the proteinuria. In the transgenic placentas, *STOX1* overexpression induces alterations of mitochondrial function and nitroso-redox imbalance, that could trigger the disease^9^.

A classical vision of the origin of the maternal symptoms in preeclampsia is through the release of toxic substances by the placenta that may be used for diagnostic and prognostic^10^, some of them such as sENG or sFLT1 being increased in the plasma of preeclamptic women even before the onset of the symptoms^11^. Preeclampsia remains, first and foremost a disease of the endothelium^12^, targeted by various placental-derived factors (antiangiogenic molecules as well as nucleic acids including miRNAs and microparticles).

Since endothelial alterations are difficult to study in human patients, we decided to evaluate thoroughly the expressional effect of a preeclamptic state on the endothelium and the heart in the STOX1 model. We used RNA-seq to examine the transcriptome of purified endothelial cells (EC) from pregnant mice bearing either WT (normal pregnancy) or STOX1 transgenic embryos (preeclamptic pregnancies). Bioinformatics analysis revealed down-regulation of genes involved in cell division, and up-regulation of genes involved in fibrosis, inflammation and cardiac hypertrophy, associated with increased heart mass, abnormal heart histology, and expression of markers of cardiac hypertrophy, consistently with the increasing number of reports describing links between preeclampsia and cardiovascular disease^13^. Overall, the alterations found in the heart and in the endothelium of wild-type females carrying transgenic embryos substantiate a model of ‘dangerous placentas’, able to induce systemic alterations in the mother. Then, we exposed an immortalized Human Umbilical Vein Endothelial Cell line (HUVEC) to plasma from human normotensive or preeclamptic pregnancies. Bioinformatics analysis revealed significant similarities between the *in vivo* alterations of the endothelium in the preeclamptic mice and the gene expression profile of HUVEC cells exposed to preeclamptic plasma. This suggests that our observations in mice could be extrapolated to women and paves the way to understand the molecular effects of preeclampsia in the cardiovascular system.

## Methods

### Animals experiments

#### Breedings and Ethics

The animals used in this study belonged to the FVB/N strain. WT ♀ mice were crossed with either WT or transgenic ♂ mice expressing the human *STOX1* under the control of the cytomegalovirus promoter, as previously described^7^. Animal experiments were carried out in strict accordance with the recommendations in the guidelines of the Code for Methods and Welfare Considerations in Behavioural Research with Animals (Directive 86/609EC). And all efforts were made to minimize suffering. All experimental protocols were approved by the Local ethics committee of Jouy-en-Josas on the Ethics of Animal Experiments of the author’s institution, INRA (Permit Number 12/035 (06.29.2012)). All animal manipulations were done according to the recommendations of the French Haut Conseil aux Biotechnologies (Permit Number N°6460 (06.25.2013)). Most of the work presented here, especially the RNA-seq analysis is focused on the TgSTOX13 mice, the only strain where homozygous transgenics could be obtained. Some of the heart expression data and heart histology were carried out conjointly on females crossed with males from the other strain available (TgSTOX42). Further details are given in the Supplementary Methods.

#### Endothelial cells purification

Six pregnant mice carrying either WT (3) or transgenic embryos (3) were sacrificed at E16.5 . Then, muscles (from forelegs, hind legs and back) were rapidly dissected, removing fat and connective tissue. EC purification procedure is detailed in the Supplementary data., In addition to validate the gene expression modifications, three additional WT mice were sacrificed and the purified endothelium was exposed during three days either to plasma of preeclamptic mice (Carrying STOX1 overexpressing embryos) or to plasma of normal pregnant mice. The plasmas were collected at day 16.5 to 17.5 of pregnancy (normal pregnancy duration is 18.5 days in the FVB/N strain). The conditions used for treatment are the same as for the human experiment described below.

#### RNA-seq analysis of RNA from endothelial cells

RNA-seq was performed by the society Genotypic Technology at Bangalore (India). The data were deposited as Fastq files as project STOX1ENDO, referred to as PRJNA275924.

#### Immunohistochemistry

Hearts were harvested, fixed in 10% formalin, and embedded in paraffin. IHC was performed as described^14^. The immunoreactivity was detected using the Novolink polymer detection system according to manufacturer's recommendations (Leica, Nanterre, France). Immunostaining was done using DAB, and tissue sections were counterstained with Mayer’s hematoxylin solution (Merck Millipore, Guyancourt, France). Anti-CTGF antibody, rabbit polyclonal HPA031074 (Sigma Prestige antibodies, Sigma, St Louis, MO) was diluted 1:100 in PBS/ 1% BSA/0.1% Triton. IHC signals were analysed using NIH ImageJ software to quantify the staining within the heart in a 20x field of view under a microscope equipped with a DC 300F camera (digital module R, IM 1000, Leica).

#### qRT-PCR

Standard conditions were utilized as described previously^9^, using a LightCycler Roche 480 thermocycler.

### Human cell experiments

#### Preeclamptic plasmas

The plasmas from patients were obtained at the Federative Research Institute 48 (Marseille, France) which approved the use of samples for the project (agreement 08-012); all the patients were duly informed of the use of their samples and gave an informed consent and the experiments (blood sampling) were carried out in accordance with the approved guidelines. Blood samples were collected in 4.5 ml, centrifuged for 10 min at 2000 g and 20 °C (Room Temperature). Plasma was aliquoted and kept at −80 °C. Details on the patients’ characteristics are given in the Supplementary Methods.

#### HUVEC and SVEC culture

HUVEC cells were grown until forming 90% confluent monolayers, washed twice in PBS and serum-starved for 12 hours. The cells were then grown in culture medium supplemented with 10% (vol/vol) plasma from either preeclamptic patients (8) or con trols (11), in 19 independent cultures. The medium was renewed every 24 hours until reaching 72 hours exposure. To note, during the experiment 5 cultures died, but no significant difference could be observed between control and preeclamptic plasmas (two from controls and three from preeclamptic plasmas, p = 0.34 by χ^2^, 1 df). The conditions for SVEC cultures were identical.

#### RNA extraction and quantitative RT-PCR conditions

Total RNA from tissues or HUVEC cells was extracted using Trizol Reagent (Invitrogen) in accordance with the manufacturer’s instructions, treated with RNase-free DNase, and quantified by spectrophotometry as described previously^7^. Reverse Transcription, and Quantitative RT-qPCR are described in Supplementary Methods, qPCR primers are listed in Table S2.

#### Microarray analysis of RNA from HUVEC exposed to control or preeclamptic plasmas

RNA quality was verified by Agilent bioanalyser 2100 and checked to have a RNA Integrity Number (RIN) systematically higher than 8. Two pools were hybridized on Agilent 8-plex 60 K microarrays at the Genomics Platform of the Cochin Institute according to standard validated protocols. The data were deposited at EMBL under the number E-MTAB-3348. Bioinformatics procedures are detailed in the Supplementary material.

### Statistical analyses

ANOVA and post-hoc Student-Neumann-Keuls t-Tests were performed for relative expression analysis in the mouse hearts using the ΔCt values method. Differences in weight in the heart were performed using ANOVA followed by Dunnett t-test. The statistics for assessing the enrichment of group of genes or TF Binding sites in gene promoter is always based on Chi2 contingency tests that are directly performed online by DAVID, STRING or Cytoscape iRegulon tool. For basic statistics, the StatistXL add-in of Excel™ was systematically used. p < 0.05 were considered significant.

## Results

### Transcriptome of endothelial cells (EC) from preeclamptic mice

EC were isolated from the limbs of pregnant females and their purity was evaluated measuring the expression of endothelial markers (Vwf^15^, Cd31^16^, Vcam1^17^ and Cdh5^18^). The enrichment of mRNA for these markers was comprised between 6.1 fold for *Cdh5* and 24 fold for *Vwf* (Supplemental Figure S1A) indicating efficient purification. The RNA extracted from the cells was subjected to Next Generation Sequencing analysis (RNA-seq), and the resulting reads were aligned along 94,818 transcripts of the mouse genome. Of these, 33,011 were validated, corresponding to 14,220 different genes (most significantly modified listed in Table S1). RT-qPCR was performed on independent EC samples to confirm the RNA-seq results (Figure S1B).

**Figure 1:**
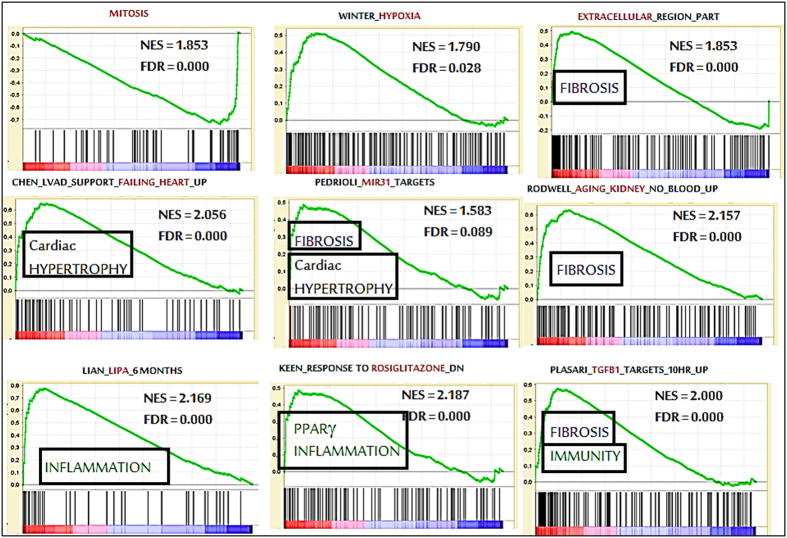
Gene Set Enrichment Analysis (GSEA). The gene expression data generated by RNA-seq was analyzed using GSEA to extract biological knowledge. Highly, significant enriched gene-sets are shown here. In every thumbnail, the green curve represents the evolution of the density of the genes identified in the RNA-seq. The False Discovery Rate (FDR) is calculated by comparing the actual data with 1000 Monte-Carlo simulations. The NES (Normalized Enrichment Score) computes the density of modified genes in the dataset with the random expectancies, normalized by the number of genes found in a given gene cluster, to take into account the size of the cluster.

The complete gene expression dataset was submitted to Gene Set Enrichment Analysis (GSEA)^19^ to extract biological knowledge. We found 212 significantly enriched gene-sets (FDR < 0.25, p < 0.01, Table S3). Critical keywords for the gene-sets were: Mitosis, Cardiac Hypertrophy, Kidney Ageing, Fibrosis and Inflammation (Fig. 1).

**Figure 2:**
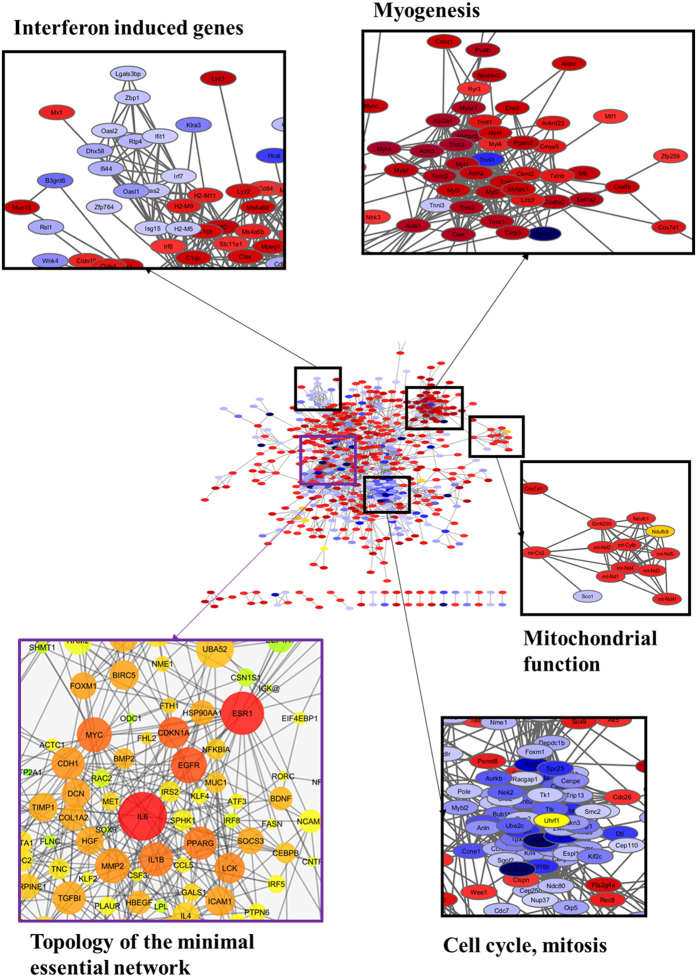
Protein-protein interaction network analysis. The DEGs identified from the RNAseq analysis, with a fold change ≥1.5 were used as seeds to construct a network of interacting proteins. The interactions between the DEGs were extracted from the STRING data base and visualized using Cytoscape software (center). The network nodes represent genes and edges interactions. The gene expression levels were mapped on the network. Red indicates up-regulation and blue down-regulation. A zoom is presented on some clusters including: Interferon-induced genes, Myogenic genes, Mitochondrial modulators and genes involved in cell cycle regulation (which are in this case drastically reduced). The left-down frame presents a topological analysis of the network. The size of the nodes is proportional to the number of connections established with other genes. The color, from green to red, is a centrality measure (betweeness centrality, BC). The BC quantifies how drastically a gene influences the structure of the whole network. IL6 is the most central gene in the network and its expression is induced in the EC by placental STOX1 overexpression.

Another method to analyse datasets of differentially expressed genes (DEGs) is to investigate if they coalesce into clusters of interacting genes/proteins. This analysis was performed with the 967 DEGs displaying a fold change (FC) ≥ 1.5. Cluster analysis by DAVID of these DEGs identified KEGG pathways such as Dilated Cardiomyopathy and Cell Cycle, consistently with the GSEA analysis (Figure S3). Then, using the STRING database and a high interaction confidence level (0.7), we detected 893 interactions among these DEGs while 413 were expected by chance (p < 10^−100^). These interactions were used to build a protein-protein interacting (PPI) network which was submitted to clustering analysis with Cytoscape to find functional modules (Fig. 2). Discrete modules were found, such as down-regulation of cell-cycle genes, and up-regulation of myogenic genes. Clusters of mitochondrial genes were also identified, consistently with data obtained from the analysis of the placenta in preeclamptic pregnancies^9^,^20^. We also detected a cluster of down-regulated genes composed of interferon targets.

Hub genes in the network were identified by topological analysis using the Cytoscape Network Analyzer. Among these, IL6, and to a lesser extent ESR1 presented the highest centrality scores. To identify transcriptional mechanisms controlling the DEGs, the top 10 % hub genes were used to build a Minimal Essential Network, MEN (Fig. 2). Promoter analysis of the MEN genes was done with the Cytoscape iRegulon apps (Figure S4, Table S4). A significant enrichment in binding sites was detected for transcription factors involved in muscle development (SOX9), inflammation (NFKB, IRF8), unfolded protein response (ATF4), cell proliferation (P53) and cell cycle (FOXM1).

### Heart weight is increased in preeclamptic mice

**Figure 3:**
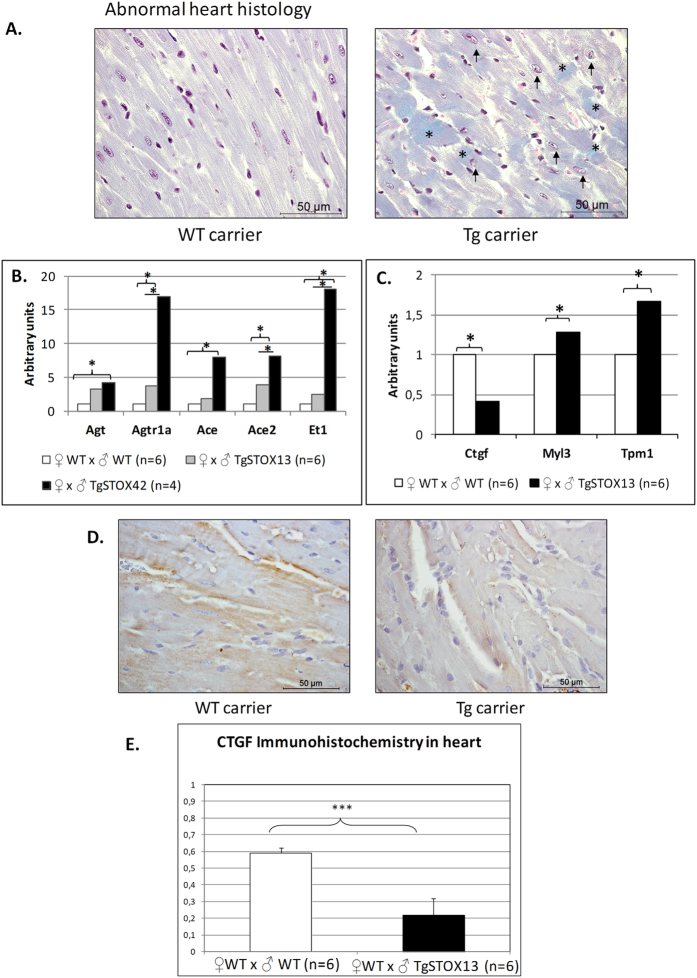
Heart histology and gene expression. Pregnant mice crossed with WT males (n = 5), transgenic TgSTO13 (n = 5) or TgSTOX42 (n = 7) males were sacrificed at the end of gestation (E16.5-E17.5) and their hearts analyzed. (**A**) Heart light-field photomicrographs of Masson's trichrome-stained sections of heart collected from dams carrying WT litters (left panel) and from dams carrying transgenic litters (right panel). Arrows indicate dilated nucleus and asterisks indicate collagen deposits. (**B**) Relative expression of the Renin/Angiotensin System and (**C**). Endothelin-1 in the heart from control versus preeclamptic pregnant mice. Hearts were retrieved at the end of gestation (E16.5-E17.5) from mice crossed with wild type males (control, white bars, n = 6) or crossed with transgenic males (preeclamptic gestation, grey and black bars). The Ct were normalized by those obtained for two reference genes, *Sdha* and *CyclophilinA*, and the expressions for the control gestations were then arbitrarily set to one. *p < 0,05. The third panel (**C**) represents the expression analysis of markers that were modified in the endothelial cells in the heart context. The genes are grouped according to their overall expression level. (**D**) IHC analysis of heart sections collected from dams carrying WT litters (left panel) and from dams carrying transgenic litters (right panel) for Ctgf staining. Representative data are from six animals in each group. Scale bar is on the right lower corner. The labeling of the protein confirmed the specific mRNA decrease of mice carrying transgenic embryos, a mark of cardiac hypertrophy. (**E**) Quantification of the IHC labeling after ImageJ treatment, transforming the image in a Black and White and measuring the labeled surfaces.

Cardiac Hypertrophy appeared systematically in the bioinformatics analyses of our dataset. Therefore, we sacrificed mice crossed with WT or transgenic males at the end of gestation, when the hypertension is the most severe, and weighted their heart. An increased weight was detected in the preeclamptic mice (Figure S3). The comparative microscopic examination revealed abnormalities in heart histology, in particular fiber disorganization, fibrosis and dilated nuclei (Fig. 3A). To assess the involvement of either hypertrophy or hyperplasia leading to this increased heart weight, we evaluated the expression of three hyperplasia markers (ki67, Topo2A and Tpx2) on heart cDNA from mice that carried either WT or transgenic embryos (6 and 10, respectively). While the expression was slightly increased for ki67 and Tpx2 (+20.7 and +21.2%, respectively) these differences were not significant (data not shown). This suggests that hypertrophy (rather than hyperplasia) is the principal mechanism responsible for the observed increase of heart weight in the preeclamptic mice.

### Altered gene expression profiles in the hearts from preeclamptic mice

We measured the expression of markers of the Renin/Angiotensin system (RAS), known as locally activated in case of heart hypertrophy^21^. We observed a significant increase of Angiotensinogen (*Agt*, which is the precursor of Angiotensin), Angiotensin type I receptor (*Agtr1a*), Angiotensinogen converting enzyme (*Ace*) and *Ace2* mRNA in WT ♀ mice previously crossed with STOX1 transgenic ♂ compared to those crossed with WT ♂ (Fig. 3B). We also measured Endothelin-1 expression in the heart from these pregnant mice (Fig. 3B), since this factor is increased in cardiac hypertrophy^22^, and is known to be induced by Angiotensin II^23^. All these genes were induced in the heart of mice carrying transgenic embryos. This was correlated with the expression levels of the transgene (about 15 times more in TgSTOX42 than in TgSTOX13).

Since the EC of pregnant mice carrying transgenic embryos revealed deregulation of genes involved in cardiac hypertrophy, we analyzed their expression in the heart. Eleven of these genes were investigated (*Ctgf*, *Dcn*, *Col1a1*, *Il6*, *Itgav*, *Dmd*, *Adcy7*, *Tnnc1*, *Myl2*, *Myl3*, *Tpm1*). We observed a significant up-regulation of *Myl3* and *Tpm1* (Fig. 3C). However the up-regulation was mild compared to that observed in EC (more than 4 fold for *Myl3*, and more than 3 fold for *Tpm1*). *Ctgf* that was induced 2.9 fold in the endothelium was on the contrary down-regulated 2.4 fold in the hearts (Fig. 3C). Ctgf immunostaining of heart sections revealed strong labeling at the edges of heart muscular fibers in the mice carrying a WT litter, whereas fainter labeling is found in the heart of the mice carrying a transgenic litter. Digitalized images quantified with ImageJ, confirmed a near 3 fold down-regulation of Ctgf protein, (Fig. 3D,E).

### Comparative analysis of ECs dysfunction in the STOX1 mouse model and in HUVECs exposed to preeclamptic plasma

**Figure 4:**
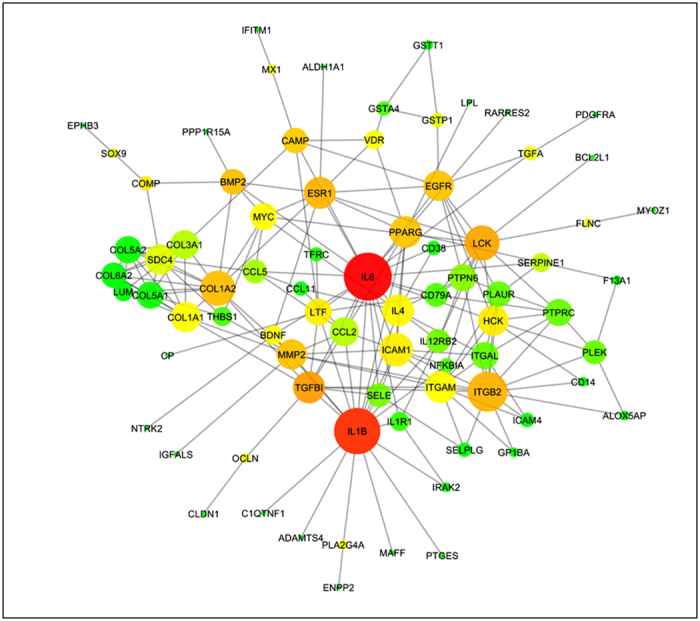
Comparison between the transcriptomic alterations of the endothelium in the STOX1 model and HUVECs exposed to preeclamptic plasma. HUVECs were exposed for 72 hours to mild or severe PE plasma and their transcriptome analyzed using human expression microarrays. DEGs identified in HUVECs exposed to mild or severe preeclampsia plasma were used to build networks (mild-PE-Ntw and severe_PE-Ntw)which were compared to the preeclamptic mice EC network, using the bioinformatics tool NeAT^24^. The figure shows the common genes and interactions resulting from the intersection between the severe-PE-Ntw and the preeclamptic mice EC network. Enrichment analysis detects sets of genes involved in inflammation, extra-cellular matrix, TGFβ cascades and coagulation processes. IL6, IL1B and ITGBI figure among the principal hub genes in the network.

To investigate the effects of preeclamptic plasma on EC, we exposed HUVECs to plasma from mild preeclampsia, severe preeclampsia or controls for 72 hours and analyzed the gene-expression profiles using human Agilent 60 K microarrays. This allowed identifying 3,863 DEGs (186 were modified more than twice, 96 up and 90 down). Non-supervised clustering analysis of the 100 genes that present the lowest p-value (computed by ANOVA) clearly discriminates between the cells treated with the control versus the preeclamptic plasmas (Supplementary Figure S5). The DEGs with a FC ≥ 1.5 were used as seeds to build two PPI networks describing the HUVECs response to mild or severe PE plasmas. We compared both networks with the preeclamptic-mice EC network using NeAT, a software which computes the union between two networks and assesses the statistical significance of their intersection^24^. The hypergeometric P-values of the intersections between the networks appeared highly significant (Table 1). However, the significance between the preeclamptic mice EC network is higher for the severe preeclampsia network than for the mild. Figure 4 shows the nodes and interactions at the intersection of these two networks. The topological analysis indicates that IL6, IL1B, TGBI, and ITGB2 are the principal hub genes (Fig. 4). Biological Processes associated with this network were « blood coagulation », « chronic inflammatory response », « leukocyte migration », and « extracellular matrix » (Table S5). Finally, to evaluate *in vitro* the effect of the plasma of mice that had had either a preeclamptic or normal pregnancy on murine endothelial cells, we prepared primary endothelial cells from three control mice, divided them into two wells from 96-well plated and applied the two types of plasma onto the two wells. Then we prepared mRNA and cDNA and analyzed the expression of a sample of genes by qRT-PCR. We also performed the same experiment on three replicates of the SVEC mouse endothelial cell line. Overall, genes induced in the RNA-seq (*in vivo experiment*) were also induced in the *in vitro* conditions, but the correlation was much stronger with primary cells (Supplementary Figure S6).

## Discussion

In this study, RNA-seq analysis revealed endothelial transcriptome alterations in the STOX1 mouse model of preeclampsia. In addition, cardiac hypertrophy and alterations of cardiac tissue were observed. However, our experimental design does not allow determining if the cardiac alterations are specifically due to the preeclamptic state induced by STOX1 or a consequence of the sustained increased in blood pressure.

The WT female mice cardiovascular/endothelial function and structure was systematically affected following foeto-placental expression of *STOX1*. The EC harbor a gene expression signature centered on IL-6 associated to inflammatory activation, a systematic down-regulation of genes involved in cell cycle and cell proliferation, and an enrichment of up-regulated genes involved in fibrosis, cardiac hypertrophy and dilated cardiomyopathy. A group of down-regulated genes composed of interferon targets was also found in line with findings showing a deregulation of circulating IFN-α in lupus-affected patients^25^ (which have a ~4 fold increase of developing a preeclampsia relative to control patients^26^). In this recent study, Andrade and co-workers showed that lupus-affected pregnant patients have a higher concentration of circulating IFNα when they develop preeclampsia than when they do not. Interestingly the authors showed that sFLT1 expression is synergistically activated by co-treatment with IFNα and sFLT1, while IFNα treatment reduces the number of endometrial tubes development. Overall the results suggest that the antiangiogenic effects of sFLT1 are sensitized by IFNα. This is consistent with several studies showing that the typeI IFN system antagonizes VEGF-stimulated angiogenesis^27^.

The IL-6 signature of inflammation in the STOX1 mouse model is clearly reminiscent of an important amount of data obtained in humans. For instance, recently, preeclampsia was associated to gene variants present in the upstream regulator of TNFα in European Americans^28^. Similarly, exposition of monocytes cultures to preeclamptic plasma induced the expression of IL-6, TNFα, and NFKB^29^, suggesting that as in humans, the pathogenesis in STOX1 mice involves aggravated inflammatory responses.

Also, comparative analysis showed that the transcriptome alterations observed in the mice endothelium are similar to that found in HUVECs exposed to preeclamptic plasma.

Down-regulation of cell-cycle genes suggests a less efficient renewal of EC, which may trigger long term alterations and ageing of the cardiovascular system. This issue may be of extreme importance to understand the reasons why women after preeclampsia are at increased risk of vascular disease as well as kidney end-stage disease, multiplied ~five-fold in women who suffered preeclampsia^30^. To note, one of the enriched GSEA gene-sets is linked to ageing kidney and fibrosis (Fig. 1). Our results are in line with a study showing that endothelial progenitor cells from PE are about twice as less numerous, with an increased differentiation time^31^.

In preeclamptic mice, heart weight was increased by ~10% and abnormal heart structure and histological features were found as well as strong alterations of the renin-angiotensin system (RAS) that may participate to cardiac hypertrophy, as observed in chronic kidney disease patients^32^. Resistance training-induced cardiac hypertrophy is characterized by up-regulation of the *AT1R* gene^21^. In return, RAS inhibition can reverse advanced cardiac remodeling by inducing aging in spontaneously hypertensive rats^33^. Endothelin-1 (ET-1) is also involved in cardiac hypertrophy induced by aging^22^. It is induced by Angiotensin II^23^. Here we have shown that several genes from the RAS system and ET-1 are increased in the hearts of mice carrying STOX1 transgenic embryos.

Cardiac hypertrophy has been described in PE women, even 6 years after the disease^34^. Pregnancy involves remodeling of heart tissue and increased heart blood flow, with molecular modifications of extracellular matrix (integrins, collagen, MMPs,..)^35^. However, this remodeling is assumed to be abnormal in preeclampsia. A link between cardiac dysfunction and preeclampsia has been substantiated by the study of Corin, a serine protease converting the pro-atrial natriuretic peptide to the active form (ANP) regulating blood pressure^36^. Mice lacking Corin have preeclampsia-like symptoms, and mutations of the gene are found in patients with preeclampsia^37^,^38^.

Interestingly, we correlated abnormal expression of Connective Tissue Growth factor (Ctgf) with endothelial dysfunction and cardiac alterations. Ctgf (also known as IGFBP-8) is a mitogen associated with renal fibrosis^39^. Induction of blood pressure overload in mice that overexpress Ctgf in the heart, demonstrated that compensatory hypertrophy was limited compared to normal littermates showing that overexpression of Ctgf tends to prevent cardiac hypertrophy^40^. Contrastingly, inhibition of Ctgf with a monoclonal antibody has also been shown to limit the remodeling of the left ventricle following increased blood pressure^41^. Herein, we show that Ctgf was induced in the endothelium but repressed in the heart. In the light of the two studies described above, we conclude that down-regulation of Ctgf in the heart co-exist with cardiac hypertrophy, consistently with the results of Gravning and co-workers (2013).

A limit of our work is that we did not have the opportunity to analyze in detail heart function in the pregnant mice (cardiac rate, ejection fraction). This could be the focus on another work less centered on global analysis of gene expression. Another limit is that we did not study the long term effects of the preeclamptic pregnancy long after the gestation, which is a major issue of the health of mothers that experienced a preeclamptic pregnancy. Clearly in a further set of experiment, the STOX1 model could be used for thoroughly assessing this question.

It has been debated whether abnormal cardiovascular function was a consequence or a predisposing cause of preeclampsia^42^. In our model, where all the female mice are WT of the same genotype, the effects that we observed are clearly a consequence of pregnancy, and not a consequence of a genetic difference between controls and affected mothers. We show that without further manipulation, STOX1 overexpression by the feto-placental unit induces cardiovascular dysfunction, suggesting that this model is a handy tool for understanding the systemic endothelial dysfunction in preeclampsia and testing therapies, to improve maternal health.

## Additional Information

**How to cite this article**: Ducat, A. *et al*. Endothelial cell dysfunction and cardiac hypertrophy in the STOX1 model of preeclampsia. *Sci. Rep*. **6**, 19196; doi: 10.1038/srep19196 (2016).

## Supplementary Material

Supplementary Information

Supplementary table S1

Supplementary table S2

Supplementary table S3

Supplementary table S4

Supplementary table S5

## Figures and Tables

**Table 1:** Comparison of the gene networks between Mouse endothelial cells and Human HUVEC cells exposed to preeclamptic plasmas.

NeAT analysis	ECs STOX1 vs mild PE	Random 1	Random 2	Random 3	ECs STOX1 vs Severe PE	Random 1	Random 2	Random 3
Expected Edges in the union	33.73	33.73	33.73	33.73	46.82	46.82	46.82	46.82
Observed edges in the union	96	4	2	3	227	10	8	4
Jacquard similarity	0.0087	0.0004	0.0002	0.0003	0.0167	0.0007	0.0006	0.0003
P Value (Hypergeometric)	8.40 10^–19^	1	1	1	1.1 10^–81^	1	1	1
