# Supplementary material for: Endothelial cell dysfunction and cardiac hypertrophy in the STOX1 model of preeclampsia
Source: Sci Rep. 2016 Jan 13;6:19196. doi: 10.1038/srep19196 (PMC4725931; doi:10.1038/srep19196)
Supplement: Supplementary Information [file srep19196-s1.pdf]

Supplementary material to

Endothelial cell dysfunction and cardiac hypertrophy in the STOX1 model of preeclampsia

Running title: Gene profiling in endothelium and heart in preeclampsia

Aurélien Ducat [1,2,3,4 #], Ludivine Doridot[1,2,3,4 #], Rosamaria Calicchio[1,2,3,4 #], Celine Méhats[1,2,3,4], Jean-Luc Vilotte[5], Johann Castille[5], Sandrine Barbaux[1,2,3,4], Betty Couderc[1,2,3,4], Sébastien Jacques[1,2,3,4], Franck Letourneur[1,2,3,4], Christophe Buffat[6], Fabien Le Grand[1,2,3,4], Paul Laissue [7], Francisco Miralles[1,2,3,4*], Daniel Vaiman[1,2,3,4*].

[#] These authors contributed equally to the experimental part of the present study.

[*] These authors contributed equally to the organization of the present study

There is no any conflict of interest declared.

This work is not submitted elsewhere.

1: Inserm, U1016, Institut Cochin, Paris, France
2: Cnrs, UMR8104, Paris, France
3: Université Paris Descartes, Sorbonne Paris Cité, Paris, France.
4 : DHU Risques et Grossesse, PRES Sorbonne Paris Cité, 53 avenue de l'observatoire, 75014 Paris.
5 : Unité Mixtes de Recherche1313 Génétique Animale et Biologie Intégrative, Institut National de la Recherche Agronomique Jouy-en-Josas, France.
6 : Genetics and Endocrine Oncology, Hôpital de la Conception Assistance PubliqueHopitaux de Marseille, Marseille, France
7 :Unidad de Genética. Grupo GENIUROS. Escuela de Medicina y Ciencias de la Salud. Universidad del Rosario. Bogotá, Colombia

Corresponding author: Daniel Vaiman
Address: U1016 INSERM, 24, rue du Faubourg St Jacques, 75014, Paris, France.
Telephone: 00 33 1 44412301
FAX: 00 33 1 44412302
Email: daniel.vaiman@inserm.fr

Words: 3800
Table: 1
Figures: 4
Supplementary Figures: 6
Supplementary Tables: 5

Legends to supplementary Tables and Figures

Figure S1: A. Validation by qPCR of the enrichment in endothelial cells (CD31+). Four endothelial cell markers were assessed by qRT-PCR after purification and compared with total cells and CD31 negative cells. Total RNA was prepared from a fraction of the non purified lysates of the muscle cells after collagenase/dispase treatment, from CD31- (flowthrough from the CD31 column not retained by the CD31-coated beads) and retained cells. The quantity of RNA was homogenized between the three subsets of cells before performing Reverse Transcirption using MMLV (Invitrogen) according to standardized protocols. B. Validation of the NGS data on a subset of 9 differentially regulated genes relevant for the paper. Six additional endothelial cells samples were purified from independent mice carrying either WT (3) or Transgenic (3) embryos. The correlation was significant ($p<0.028$ in a two directional test carried out using Vassarstat (http://vassarstats.net/rsig.html)).

Figure S2: Two examples of KEGG pathways significantly enriched in the RNA-seq dataset of DEG observed between endothelial cells from mice carrying either WT or STOX1-transgenic embryos. Each star corresponds to a gene that was modified in the dataset. The upper part shows genes modified in Dilated cardiomyopathy. In this case they were all up-regulated. On the contrary for the Cell-cycle pathway, illustrated in the lower part of the figure, the genes were down-regulated. The p values for the enrichment in genes involved in these pathways were evaluated by DAVID and were <0.0001.

Figure S3: Hearts have an increased weight in female mice carrying transgenic embryos. While we had a trend with the TgSTOX13 embryos (4.5 mg more) it was significant with the TgSTOX42 embryos (12mg more, corresponding to a ~10% increase).

Figure S4: iRegulon analysis of promoters of genes modified in the RNA seq experiment. iRegulon, a add-in to the Cytoscape program, was parametered to find Transcription Factor Binding Sites (TFBS) in the 20 kb centered around the Transcription Start Site (TSS) of the genes belonging to the Minimal Essential network of genes identified in the RNA-seq experiment. Genes in red are induced while genes in blue are repressed. FOXM1 was found are mildly down-regulated (40% less expression). It is a major regulator of cell-cycle and its down-regulation could contribute to explain the deregulation of cell cycle genes that we observed in the endothelial cells. IRF8, by contrast is up-regulated and can contribute to the regulation of genes involved in inflammation and in the Interferon cascade. SOX9, which is also up-regulated (in the lower part of the figure) could play a role in the up-regulation of genes involved in myogenic function, and thus contribute to explain the cardiac hypertrophy that was observed in the mice and in the expressional network.

Figure S5: Non-supervised clustering analysis on the 100 most significant genes clearly discriminates cells exposed to control from those exposed to preeclamptic plasma. Similar heatmaps were also obtained when the number of genes was increased to 200, 1000 and 2000 genes.

Figure S6: The expression of 7 genes was analyzed by q-RT PCR on SVEC (three replicates) or primary EC (three independent mice), cultivated during three days with 10% of plasma from pregnant control or pregnant preeclamptic mice. After RNA extraction and q-RT PCR and normalization using two reporter genes (SDHA and Cyclophilin-PPIA), the expression levels were plotted. There was an excellent correlation between the RNA seq data and the effect of the treatment on primary EC cells. The correlation was less strong with the SVEC cell line. For the HUVEC experiment,we do not have the information for SERPINE1 in the array; nevertheless the correlation was relatively high ($R2 = 0.29$, $R= 0.53$, not shown in the figure.

Table S1: List of the 100 top genes modified in the endothelium following the RNAseq experiment. In this case, compared to the other analyses each gene is significant by itself. Despite their small number, clustering by STRING revealed strongly enriched Protein interaction networks (314 interactions observed, 69 expected, $p< 10^{-100}$). The KEGG enrichment revealed the following GO:

GO_id	Term	NumberOfGenes	p-value	p-value_fdr
4510	Focal adhesion	11	1.14E-9	3.24E-7
4610	Complement and coagulation cascades	7	3.37E-8	4.79E-6
4512	ECM-receptor interaction	6	2.02E-6	1.91E-4
4260	Cardiac muscle contraction	4	2.92E-4	1.67E-2
4810	Regulation of actin cytoskeleton	6	2.94E-4	1.67E-2
4921	Oxytocin signaling pathway	5	5.04E-4	1.95E-2
4974	Protein digestion and absorption	4	5.6E-4	1.95E-2
5322	Systemic lupus erythematosus	4	6.09E-4	1.95E-2
4145	Phagosome	5	6.17E-4	1.95E-2
5144	Malaria	3	8.6E-4	2.44E-2
5150	Staphylococcus aureus infection	3	1.25E-3	3.22E-2
5146	Amoebiasis	4	1.6E-3	3.79E-2
4670	Leukocyte transendothelial migration	4	1.76E-3	3.84E-2
4611	Platelet activation	4	2.22E-3	4.5E-2
5205	Proteoglycans in cancer	5	2.41E-3	4.52E-2
4530	Tight junction	4	2.55E-3	4.52E-2
4151	PI3K-Akt signaling pathway	6	3.35E-3	5.39E-2
5133	Pertussis	3	3.42E-3	5.39E-2
5412	Arrhythmogenic right ventricular cardiomyopathy (ARVC)	3	3.84E-3	5.74E-2
5410	Hypertrophic cardiomyopathy (HCM)	3	5.29E-3	7.52E-2
5414	Dilated cardiomyopathy	3	6.23E-3	8.42E-2

This shows that this small subset of genes is overall enriched similarly in pathways than the more complete dataset analyzed without threshold by GSEA.

Table S2: RT-q PCR primers used throughout the study.

Table S3: Gene pathways identified as enriched by GSEA using the genes modified in the endothelial cells of mice carrying STOX1 embryos. The pathways are grouped by colors according to the most relevant keywords identified. To note, there are 6 gene-sets involved in heart function and disease, 64 associated to immunological signatures, 12 associated to inflammation, 16 to muscle function, development and disease, 27 to cell proliferation, 12 to oxidative stress and 10 to vascular function. The interpretation of the non-explicit heads of the column are the following: SIZE correspond to the number of genes of the dataset that are also in the reference dataset. ES and NES are Enrichment Score and Normalized Enrichment Score. A NES of 2 means that there are twice as many genes in the dataset study that expected by mere chance. This allows to calculate a p –value (NOMinal p-value) a FDR q-value and a FWER q-value which is more stringently used to limit the rate of type 1 in multiple testing and aiming at reducing the probability of even one false discovery, rather than the expected number of false discoveries (given by the FDR q-value). The “RANK at MAX” column refer to the ranking of the last gene contributing to the enrichment in the GSEA procedure Since 14000 genes were identified it means that for instance the gene set LIAN_LIPA-Targets (line 78), 41 genes belonged to the gene set, and the last to contribute to the NES is the 905th. Finally, tags, lists and signal are indication of the presence and distribution of the positive genes in the dataset is the percentage of gene hits in the reference dataset. Details are available at http://www.broadinstitute.org/gsea/doc/GSEAUserGuideTEXT.htm

Table S4: Listing of the iregulon output for the analysis of the minima network of genes described as Supplemental Figure 4. NES is defined in table S3 legend (for instance, HP1B3

binding site is found 6.699 fold more frequently than expected by random in the promoters of genes belonging to the MEN. AUC represents the Area under the curve, similar to the contribution of each transcription factor motif to the complete dataset.

Table S5: NeAT output for gene sets belonging to the transcriptome of HUVEC exposed to preeclamptic plasma as well as to the endothelium of mice carrying STOX1 transgenic embryos. In blue are all the significant genesets at a FDR < 0.05.

Endothelial cell dysfunction and cardiac hypertrophy in the STOX1 model of preeclampsia

By Ducat et al.

Supplementary Methods

Patient characteristics

	Control group (n =10)	preeclampsia group (n=10)	p value
Maternal age (years)	30	28	ns
weight in early pregnancy (kg)	63	60	ns
Liveborn infants	10	10	
Fetal gender			ns
Male	5	6	
Female	5	4	
Gestational age group			P=0.001
2nd trimester	0	5	
3rd trimester	10	5	
arterial pressure mm Hg (systolic - diastolic)	115/85	168/93	$P=5.10^{-15}$
Proteinuria (g/l / 24h)	<0.5	2.8 g/l/24h	$P=1.9\ 10^{-5}$

Value presented as median (or number)

Animals

Animals (FVB/N) were bred in the animal facility of INRA (Jouy-en-Josas, France) in a controlled environment (light/dark cycle, temperature, free access to food and water), according to standard procedures for manipulating animals by experimented staff under authorization of the Police Prefecture ("Service de la Protection et Santé Animale et de la Protection de l'environnement" authorization RL-0801432-30801038, during all the experiments all the efforts were made to set animal suffering to the minimum). The females were inspected daily for vaginal plugs. Detection of a

vaginal plug in the morning was designated as day 0.5 of pregnancy (E0.5 or 0.5 dpc). Litter sizes were systematically recorded immediately after birth. A total of 20 mice (of different crosses) were sacrificed at the end of gestation using standard accepted procedures after anaesthesia (Xylazine/Ketamine) (E16.5-E17.5) and their hearts were weighted and collected (half was kept in PFA for histological analysis, half was placed in Trizol for RNA extraction, see below). Muscles from the limbs were also collected for endothelial cells purification.

RNA extraction and quantitative RT-PCR conditions

Reverse transcription (RT) was carried out according to a standardized protocol. Briefly, 4 µg of total DNase-treated RNA was reverse transcribed in a volume of 25 µL at 39°C using the M-MLV Reverse Transcriptase (Invitrogen) and random primers during 1 hour.

Quantitative RT-PCR was carried out using the LightCycler® 480 SYBR Green I Master Mix (Roche Applied Science) in accordance with the manufacter's instructions. The reaction was performed in a Light-Cycler 480 Thermocycler (Roche Applied Science). Primers were designed for the coding sequences of the different genes using the PRIMER3 software (http://frodo.wi.mit.edu/cgi-bin/primer3) and aligned with basic local alignment search tool software (BLAST) to avoid nonspecific annealing. Samples were submitted to cycling according to the following PCR program: 95°C for 5 min followed by 45 cycles of 3 temperature steps (94°C for 10 s, 58°C for 15 s, and 72°C for 15 s). Finally, samples were submitted to a progressive temperature elevation (from 65 to 99°C at 0.1°C/s), resulting in a melting curve, enabling to check the PCR products homogeneity. In addition, amplification products were systematically controlled by agarose gel electrophoresis. The threshold cycle number (Ct) values were collected with the LightCycler480 software (Roche Applied Science) in the exponential phase of the PCR reaction. These Cts were normalized by the Ct values obtained for the murine succinate dehydrogenase subunit A (*Sdha*) and the cyclophillin A (*CycloA*) used as normalising genes. All primers are presented as Supplemental Table 2.

Bioinformatics

Bioinformatics analysis was used combining ArrayMining software (http://www.arraymining.net/ [14]) and the Cytoscape package. The comparative analysis of networks to

examine the links between these experiments and the RNA-seq of endothelial cells was carried out using NeAT [15]. Basically, the software computes the edges (interactions) that are common to both networks or found in only one of the two networks. Then it calculates a Jaccard coefficient (% of edges at the intersection), the number of edges expected by chance at the intersection, and a hypergeometric P-value (corresponding to the probability of finding x edges at the intersection when y are expected by chance). In a second step, one of the two networks is randomized and a new comparison is done to calculate the random expectation. Analysis of the transcriptome for RNA-seq and microarrays was carried out using three complementary approaches: DAVID analysis of the most deregulated genes, GSEA analysis of enriched pathways and Cytoscape analysis following the identification of networks of modified genes defined by STRING http://string-db.org/newstring_cgi/show_network_section.pl. The analysis was carried out by pooling the different transcripts FPKM values to take into account the global values per gene (all transcripts) or by using the differential transcripts only. Overall network analysis gave very similar results, and thus only the first analysis, considered to represent the most complete transcript information is presented here.

A.

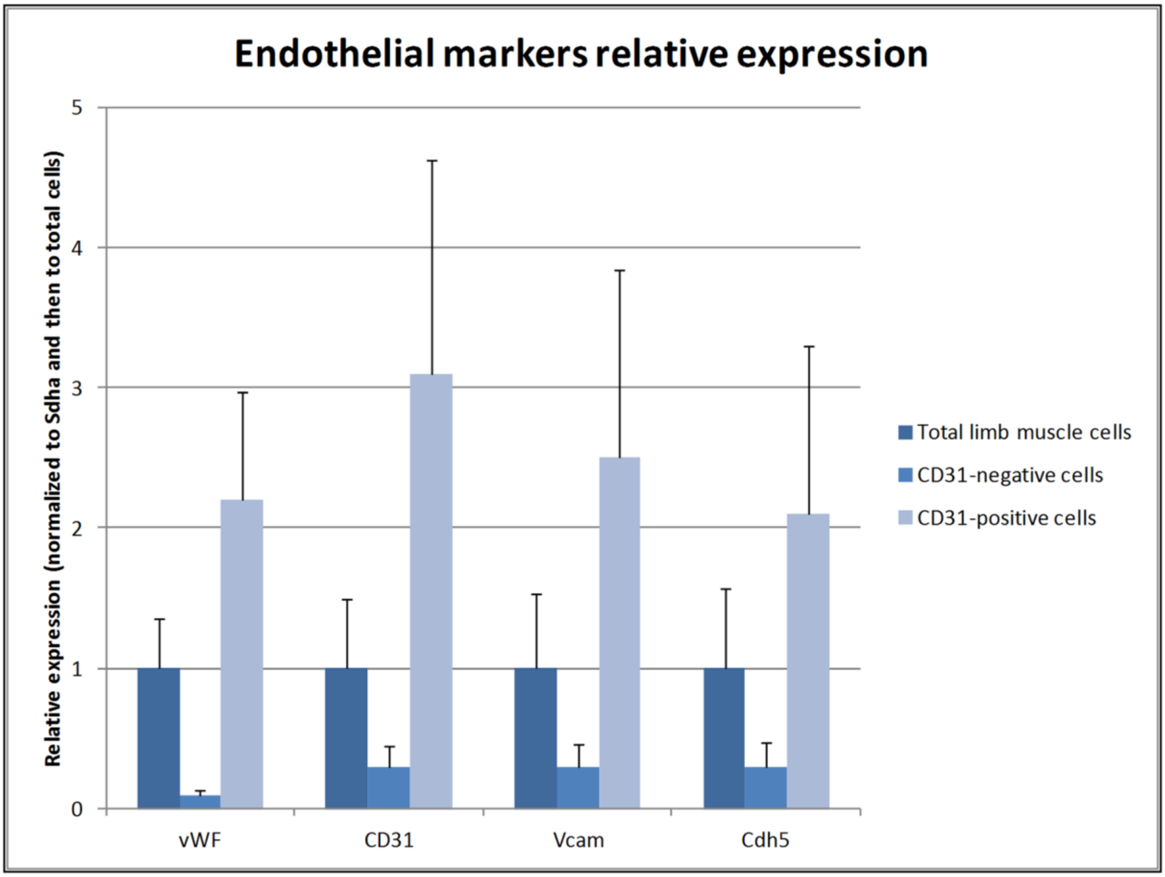

B.

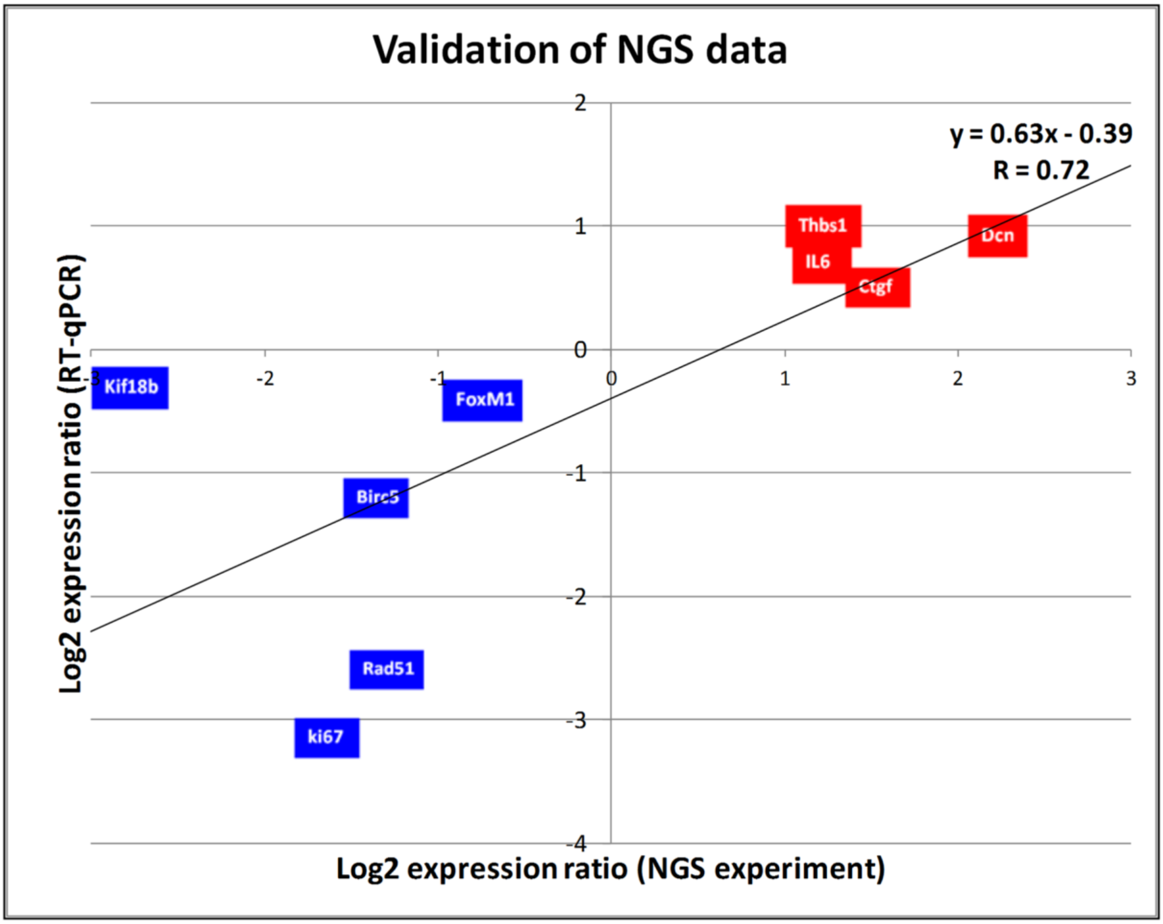

Ducat et al, Supplemental Figure 1

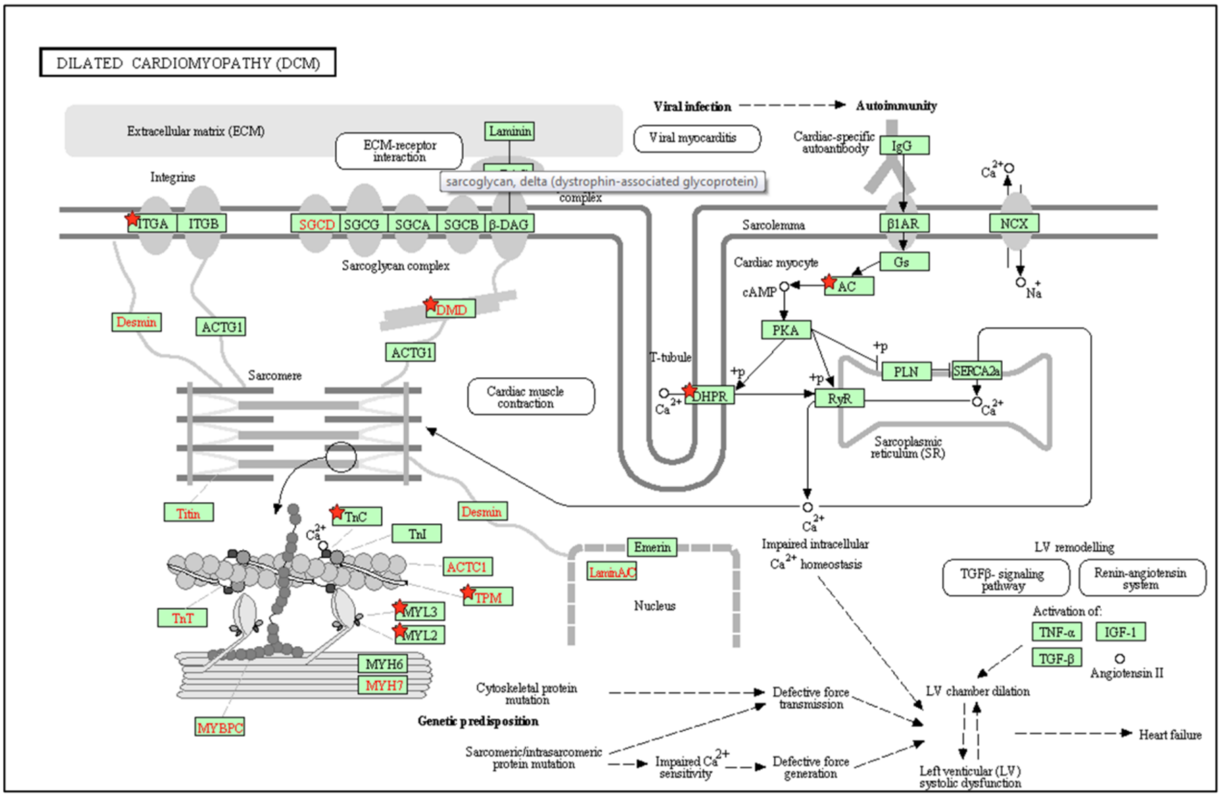

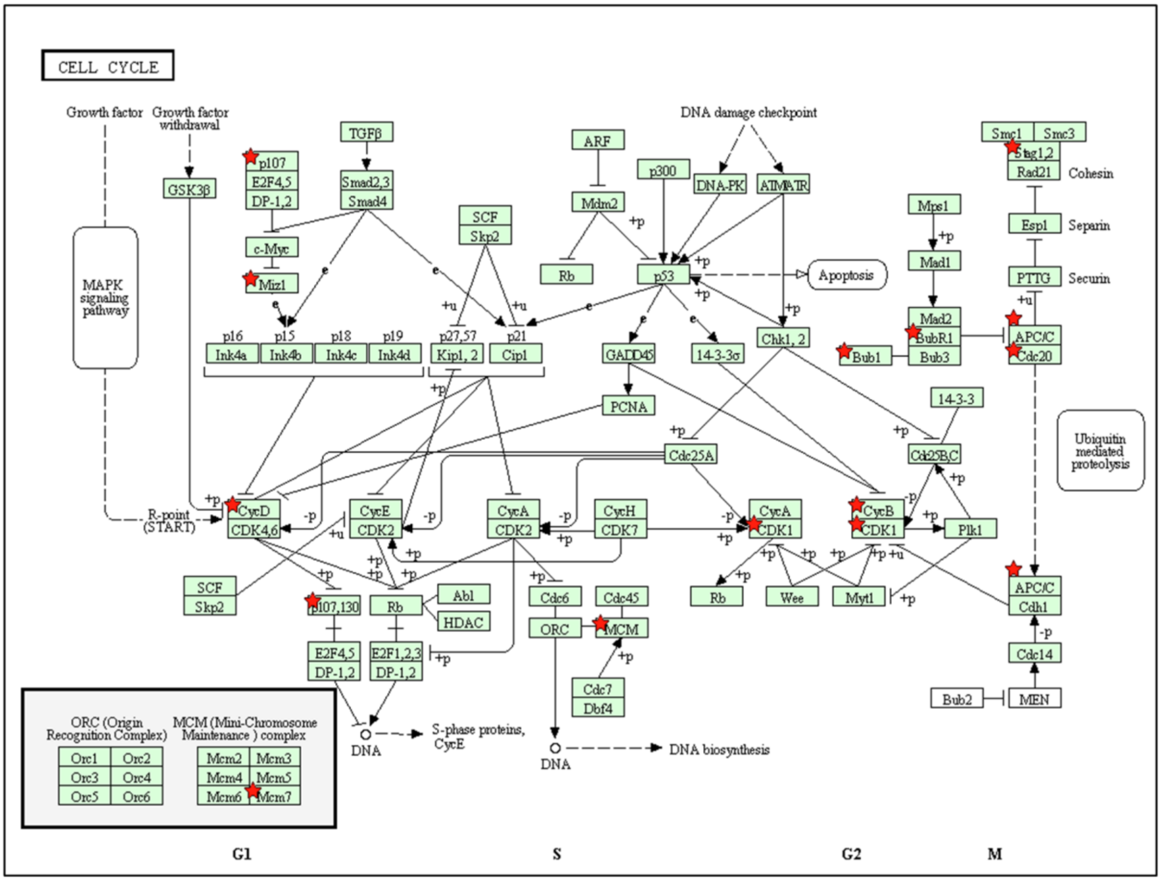

Ducat et al, Supplemental Figure 2

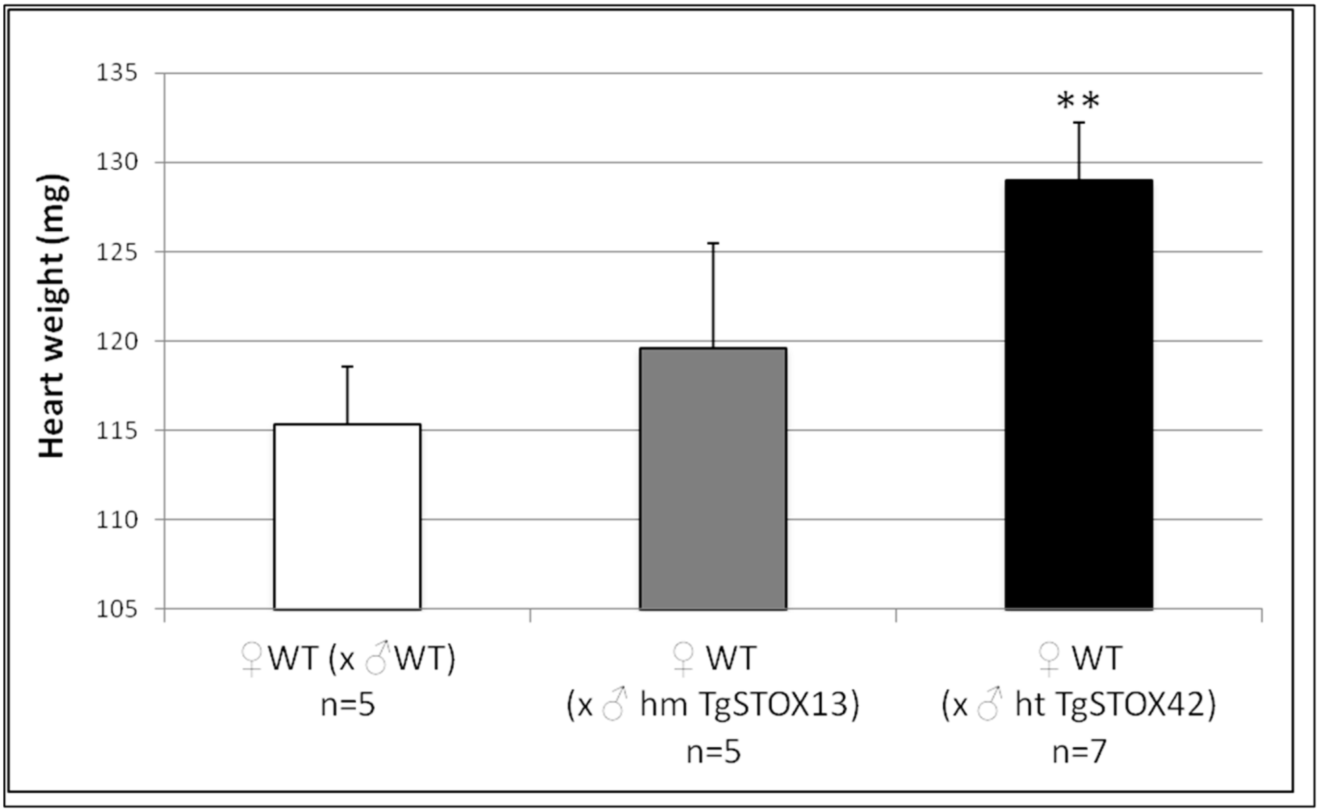

Ducat et al, Supplemental Figure 3

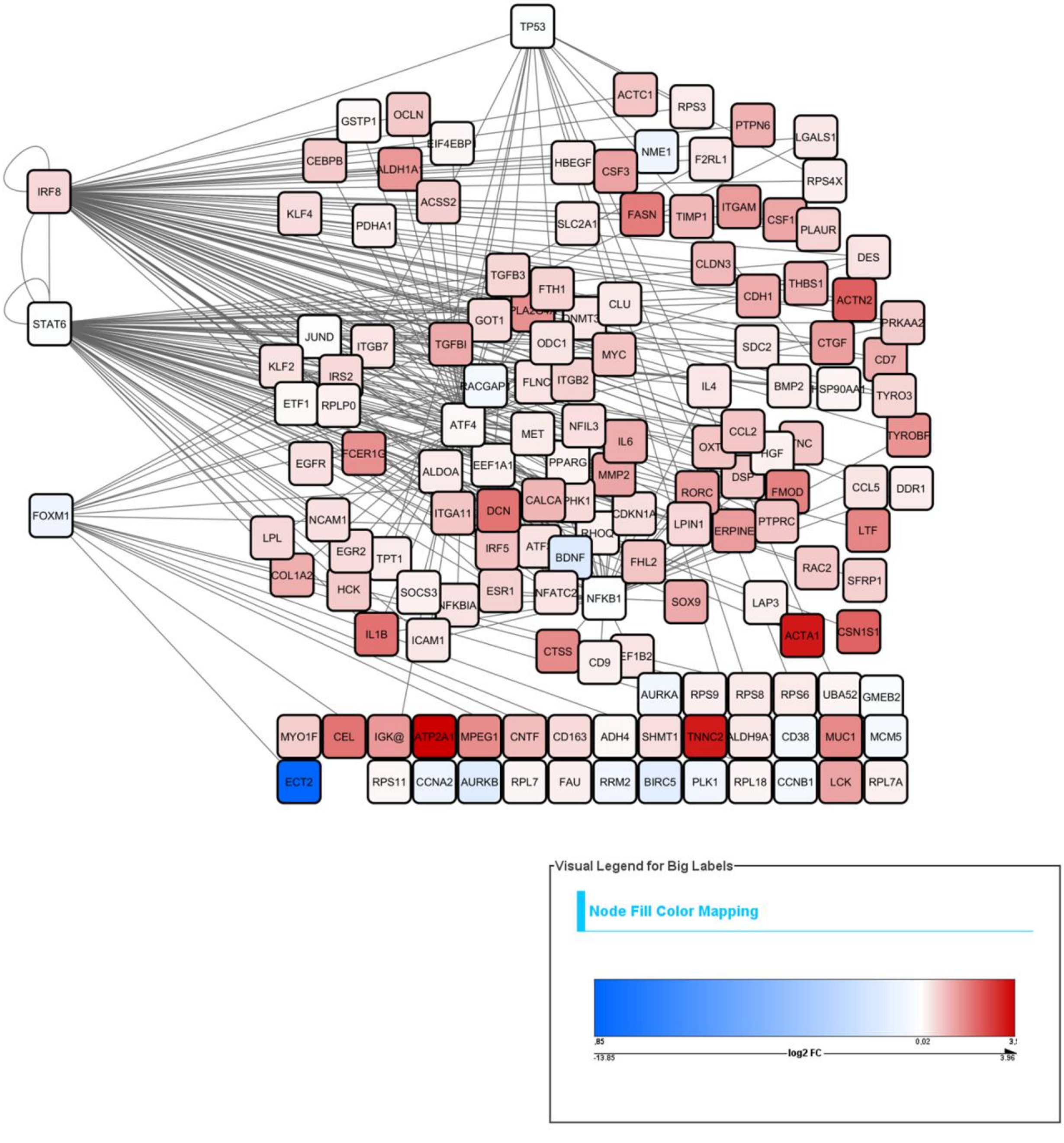

Ducat et al, Supplemental Figure 4

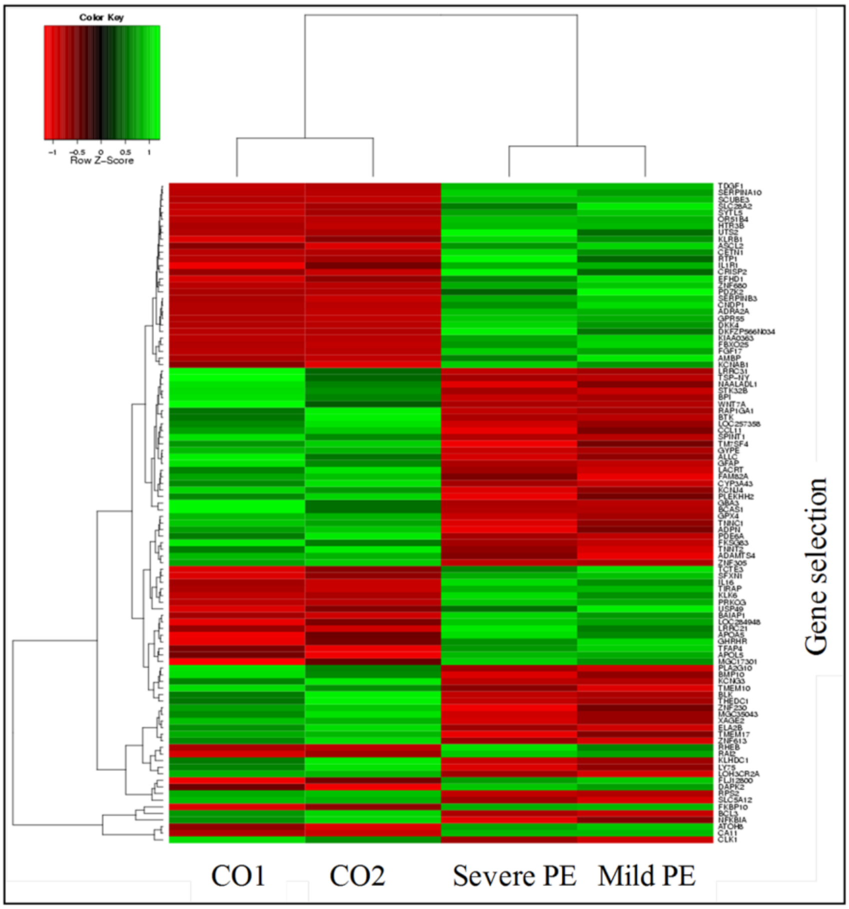

Ducat et al, Supplemental Figure 5

Correlation between the RNA seq experiment and the effects of Plasma exposure (from WT or transgenic pregnancies on endothelial cells)

RNA-seq	SVEC	Primary EC	Gene name
1,62	0,63	2,27	**Aldh1a1**
1,79	1,14	1,69	**Htra3**
1,17	0,52	1,97	**Ifitm1**
-0,77	0,46	-1,60	**Cdkn1c**
1,05	-0,24	-0,04	**Cpxm2**
0,63	0,99	1,23	**Efhd1**
1,54	0,44	2,43	**Serpine1**

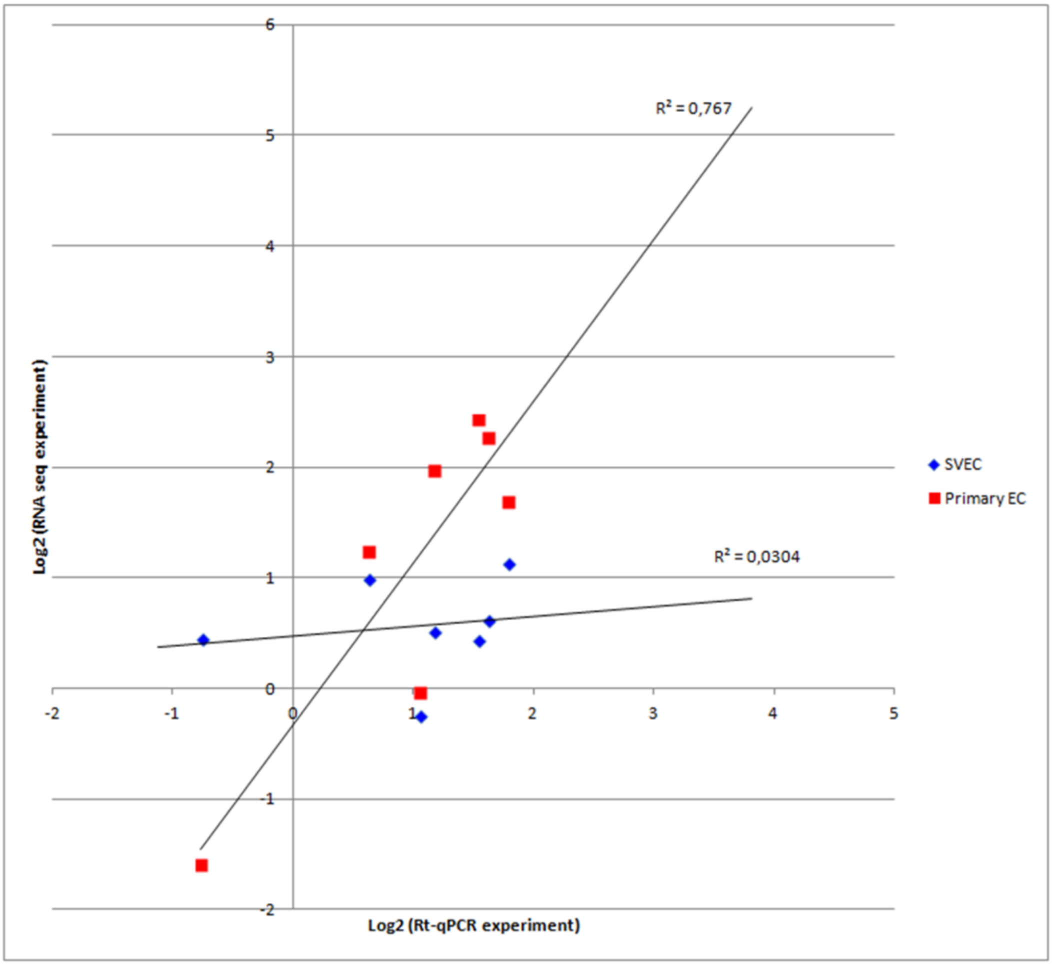

Ducat et al, Supplemental Figure 6
